# Impact of Coagulase-Negative Staphylococci in Mixed Intramammary Infections with Streptococci on Milk Quality

**DOI:** 10.3390/biology14121672

**Published:** 2025-11-25

**Authors:** Sho Nakamura, Sophorn Nouv, Kanan Dim, Sambo Na, Panhavatey Sokhom, Shuichi Matsuyama, Tetsuma Murase, Satoshi Ohkura, Witaya Suriyasathaporn

**Affiliations:** 1 Laboratory of Animal Production Science, Graduate School of Bioagricultural Sciences, Nagoya University, Nagoya 464-8601, Japan; shonakam@agr.nagoya-u.ac.jp (S.N.);; 2 Cambodia Campus, Asian Satellite Campuses Institute, Nagoya University, Nagoya 464-8601, Japan; 3 Department of Animal Health and Veterinary Public Health, General Directorate of Animal Health and Production, Phnom Penh 120603, Cambodia; 4 Faculty of Veterinary Medicine, Royal University of Agriculture, Phnom Penh 120501, Cambodia; 5 Laboratory of Veterinary Theriogenology, Faculty of Applied Biological Sciences, Gifu University, Gifu 501-1193, Japan; 6 Faculty of Veterinary Medicine, Chiang Mai University, Chiang Mai 50100, Thailand; 7 Research Center of Producing and Development of Products and Innovations for Animal Health and Production, Chiang Mai University, Chiang Mai 50100, Thailand

**Keywords:** milk composition, somatic cell count, mastitis pathogens, farm management

## Abstract

This study investigated the relationship between milk composition among different mastitis pathogens. The study was conducted from January to February 2025 on three different dairy farms in Cambodia, utilizing 241 crossbred Holstein–Friesian milking cows. Quarter milk samples were aseptically collected from randomly selected milking cows, mostly in mid- and late lactation, including 450 samples from 3, 25, and 213 cows and 11, 88, and 351 quarters from Farms A, B, and C, respectively. The milk samples were analyzed for bacterial identification, somatic cell count, and milk composition. Mastitis pathogens were grouped primarily based on their genus, including streptococci, coagulase-negative staphylococci (CNS), Gram-negative bacteria (Gram-negative), CNS plus streptococci (Mixed), and those with no detected bacteria (None). Pearson’s correlation coefficients were used to determine the correlations between milk composition and somatic cell scores (SCSs). The comparison of milk composition and SCS means across bacteria groups, farms, and intramammary infection (IMI) status was performed using repeated linear models. Significance was defined as *p* < 0.05, and a tendency was defined as *p* < 0.10. The results show that the collected quarter milk had a low fat concentration. Milk composition and SCS were associated with the farm (*p* < 0.05). Regarding bacterial groups, the SCS of milk with streptococci was higher than that of milk with CNS and non-IMI milk (*p* < 0.05). Milk with a mixed IMI of streptococci and CNS had significantly lower lactose and solid-not-fat (SNF) levels than milk with CNS or Gram-negative bacteria. In contrast, the freezing point of the mixed IMI was higher than that of CNS and non-IMI milk (*p* < 0.05). In conclusion, the compositions of milk from CNS IMI did not deviate from those of milk from healthy quarters; however, the mixed infection of CNS and streptococci caused lower milk quality. These findings underscore the importance of CNS IMI, which can lead to significant economic losses in the case of infection with other pathogens. However, as this study employed a cross-sectional design, which is suitable for identifying associations rather than establishing causation, further studies are needed.

## Simple Summary

Mastitis caused by bacterial infections within the mammary glands severely compromises milk quality in dairy cattle. Alarmingly, there has been a rising trend in mixed infections, which underscores the urgent need for improvements in existing mastitis control programs. This study compellingly demonstrates that mixed infections involving both coagulase-negative staphylococci (CNS) and streptococci result in a more significant decline in milk quality than infections with either pathogen alone. Therefore, it is essential for mastitis control strategies to incorporate measures against mixed infections in order to safeguard the quality of milk.

## 1. Introduction

Milk is a nutrient-dense food that plays a vital role in human health, particularly by supporting growth and development in children. Cambodia imports USD 56.3 million worth of milk, making it the 28th largest milk importer globally, which leads to high local prices. In response, the government is encouraging local dairy production, with some farms supplying major supermarkets in Phnom Penh since 2022. However, there remains a lack of healthcare products, services, and knowledge related to dairy cattle in the country. Regardless of the country, ensuring the highest quality of raw milk in the dairy industry is essential to maximize its nutritional and health benefits. Among the factors influencing milk quality, bovine mastitis is one of the most prevalent and economically important diseases worldwide. It is associated with reduced milk yield, altered milk composition, elevated somatic cell counts (SCCs), and substantial economic losses to the dairy industry [1,2,3].

Mastitis can be caused by a wide range of pathogens, including *Streptococcus* spp., *Staphylococcus* spp., and various Gram-negative bacteria [4,5]. *Streptococcus* spp., especially *S. agalactiae*, are well known contagious and environmental pathogens that are strongly associated with deteriorated milk quality and elevated SCC [6]. In contrast, coagulase-negative staphylococci (CNS), previously known as minor or opportunistic pathogens, are often associated with subclinical mastitis and mild intramammary infections (IMIs) [7], but do not significantly affect milk yield or composition [8]. However, various studies have indicated that CNS have emerged as significant contributors to udder health issues, as they are now among the most frequently isolated pathogens in subclinical mastitis [9,10]. Furthermore, our recent study showed CNS to be implicated in persistent IMI, causing a high rate of mastitis on a farm with a limited mastitis control program [11]. Some species of CNS, such as *S. chromogenes*, were able to persist and transmit within and between cows [11]. A study examining the coculture of mastitis pathogens in milk reveals that the growth rate of *S. uberis* was higher when cocultured with *S. chromogenes* than when it was cultured alone [12]. These may alter the composition of milk during mixed infections.

Although the individual impact of CNS on milk quality has been considered to be limited, evidence suggests that coinfections with other mastitis pathogens may exacerbate adverse effects. Cows with mixed intramammary infections showed fluctuating levels of cytokines, indicating an extra response of the udder defense mechanism [13]. In dairy goats, intramammary infection with CNS did not affect milk yield, whereas infection with *Corynebacterium bovis* was associated with increased milk yield [14]. In particular, mixed infections involving CNS and *Streptococcus* spp. have been hypothesized to produce more pronounced alterations in milk composition, including decreased lactose and solid-not-fat (SNF) content and increased SCC [15]. Despite this, few studies have directly compared the effects of CNS, *Streptococcus* spp., and their mixed infections on milk quality traits under field conditions.

The objective of this study was to evaluate the impact of CNS in mixed IMI with Streptococci on the changes in milk composition and SCC. Specifically, we aimed to clarify the role of CNS in coinfections and their contribution to reduced milk quality by comparing milk quality parameters from quarters infected with CNS, *Streptococcus* spp., mixed infections, and non-infected controls across three commercial dairy farms.

## 2. Materials and Methods

This study was conducted from January to February 2025, utilizing three dairy farms: a training smallholder dairy farm (Farm A), a local private medium-sized farm (Farm B), and a private large and intensive farm (Farm C). Farm A and Farm B had averages of 10 and 30 crossbred Holstein–Friesian milking cows, respectively, imported from Thailand, while Farm C had an average of 700 lactating purebred Holstein–Friesian cows from Australia. All cows were housed in a free-stall facility with concrete floors for Farms A and B, but with a soil floor in Farm C. The feed management at Farms A and B was based on recommendations from local veterinarians, primarily utilizing locally available roughages, with concentrates mixed from available feed grains, molasses, mung bean, soybean, rice bran, and cassava pulp. For Farm C, the farm’s foreign dairy experts recommended feed management in accordance with NRC (2001) [16]. Since the dairy industry in Cambodia has only been established for several years [11], Farms A and B utilized local animal medicines, particularly those commonly used for other livestock. In contrast, Farm C imported chemicals and medicines for dairy cattle from other countries. The milking instruments used at Farms A, B, and C included a 2-bucket system, a pipeline with a semi-automatic instrument, and a fully automatic instrument, respectively. Routine milk conductivity testing was performed for monitoring of mastitis at Farm C. The California Mastitis Test was partially used at Farms A and B for the diagnosis of subclinical mastitis, and systemic antibiotics were used for mastitis treatment. The average rolling milk production for Farms A, B, and C was approximately 5–10, 10–15, and 15–20 kg/cow/day, respectively.

This cross-sectional study was conducted with lactating cows, mainly in the mid- and late lactation stages, across the farms’ lactation group arrangements. All lactating cows at Farms A and B were included, with all cows at Farm A and most at Farm B in the mid- and late lactation stages. At Farm C, cows in the mid- and late lactation group arrangements were randomly selected. Trained veterinarians aseptically collected quarter milk samples after discarding the first few streams of foremilk. Approximately 1–5 mL of milk from each quarter was collected into sterile tubes, stored at 4 °C, and transported on ice to the laboratory at the Cambodian Campus of Asian Satellite Campuses Institute (Nagoya University), located at the Royal University of Agriculture in Phnom Penh, Cambodia, within two hours for analysis. For bacterial examination, 10 µL of each milk sample was streaked onto 5% bovine blood agar plates and, when necessary, MacConkey agar was used to detect Gram-negative bacteria. Plates were incubated at 37 °C for 24–48 h. Colonies were evaluated for morphology, and samples showing more than three distinct colony types were regarded as contaminated and excluded. A quarter was defined as having an IMI when ≥1000 cfu/mL of one or two pathogens was isolated from a single sample [17,18]. For bacterial identification, catalase, coagulase, and CAMP tests were performed following the National Mastitis Council (NMC) guidelines [19]. *Staphylococcus* spp. were identified by a positive catalase reaction (bubble formation) and further differentiated using a coagulase test with diluted plasma; clot formation within 24 h indicated *S. aureus. Streptococcus* spp. were catalase-negative, and *S. agalactiae* was confirmed by a positive CAMP test showing an arrowhead-shaped hemolytic zone. Milk composition (fat, protein, lactose, solid-not-fat, total solids, and freezing point) was analyzed on the same day using an FT-IR analyzer (MilkoScan^TM^ Mars, FOSS, Hilleroed, Denmark). The rest of the milk samples were kept at −20 °C until the somatic cell counts (SCCs) were measured using an automated cell counter, the NucleoCounter^®^ SCC-100™ (ChemoMetec, Allerod, Denmark) according to the manufacturer’s instructions, with a 1:1 mixture of milk and reagent (50 µL each).

### Statistical Analysis

All milk composition parameters were initially assessed for normal distribution using a histogram. Means with standard error of the mean (SEM) and their ranges are used as descriptive statistics for continuous variables, while percentages are used to describe discrete variables. The values above the measuring range or limit of quantitation of the instrument, including 0–48% fat, 0–6% protein, 0–50% TS, 0–12% SNF, and 0–6% lactose, as specified by the manufacturer, were excluded. To minimize the influence of extreme non-normal values [20], some modifications were made according to the data: a 5% trimmed mean was calculated to remove the top 5% or bottom 5% of observations from a right- or left-deviated dataset, respectively. The remaining 95% of the data was then used for the final analysis. Due to their non-distributed data, the SCCs were logarithmically transformed to somatic cell scores (SCSs) using the following formula:

$${SCS=log}_{2}\left(\frac{SCC}{100,000}\right)+3,$$

where SCC values are given in cells per milliliter [21]. Mastitis pathogens were grouped primarily based on their genus, including Streptococci, Staphylococci excluding *S. aureus* or CNS, Gram-negative bacteria (Gram-), mixed bacteria (Mixed), and those with no detected bacteria (NO). Intramammary infection status (IMI = yes) was defined as the presence of at least one bacterium in the milk sample. Pearson’s correlation coefficients were calculated to determine the correlation among continuous variables, including fat, protein, lactose, solid-not-fat, total solids, freezing point, and SCS. Fisher’s exact chi-square test determined comparisons of percentages of mastitis bacteria groups among the farms. The comparison of means for each milk composition and SCC across bacteria groups, farms, and IMI status was performed using a repeated linear model (Proc Mixed, SAS OnDemand for Academic (ver. 9.4)). The cow factor was defined as the cluster factor, and a compound symmetry correlation structure was applied for the nested quarters within each cow. Least square mean calculations were used to compare the groups. Significant levels were defined as *p* < 0.05, and a tendency was defined as *p* < 0.10. All model fittings were evaluated and met the assumptions for residual diagnostics of normality and homoscedasticity. As is common in large datasets where the test can detect trivial and practically unimportant deviations from normality and given the known robustness of mixed models to mild violations of normality, the high W-statistic (0.95), the diagonal line of the Q-Q plot, and the symmetric bell-shaped histogram of residuals indicate a near-perfect fit. Therefore, normality was considered to be adequately met. For homoscedasticity, the residual and predicted values were plotted, and the pattern was accepted when the cloud of points was evenly scattered around the zero line.

## 3. Results

**Figure 1:**
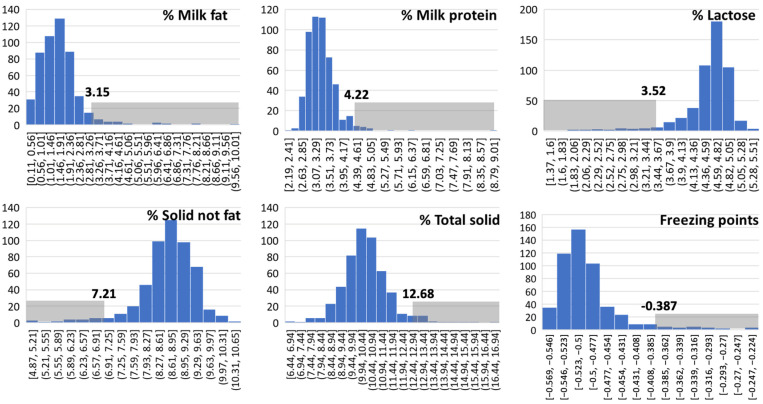
Data distribution of percentages of milk fat, protein, lactose, solid-not-fat (SNF), total solids (TS), and freezing points (FPs) of bovine quarter milk from each quarter. The gray boxes indicate the excluded data, which are the 5% trimmed areas defined by the 5th or 95th percentiles, depending on the skewed side. The numbers above the gray boxes were the 5th (left-side) or 95th (right-side) percentiles.

The ranges of %fat, %protein, %lactose, SNF, TS, and FP were 0.11–9.95%, 2.19–8.89%, 1.37–5.39%, 4.87–10.63%, 6.44–18.66%, and −0.569 to −0.237, respectively. Histograms of the milk compositions, along with the top and bottom trimmed points, are shown in Figure 1. After excluding the trimmed data, the %Fat and TS values of the quarter milk were relatively low, ranging from 0.11% to 3.15% and from 6.44% to 12.68%, respectively. In contrast, the FP was relatively high compared to normal milk.

**Figure 2:**
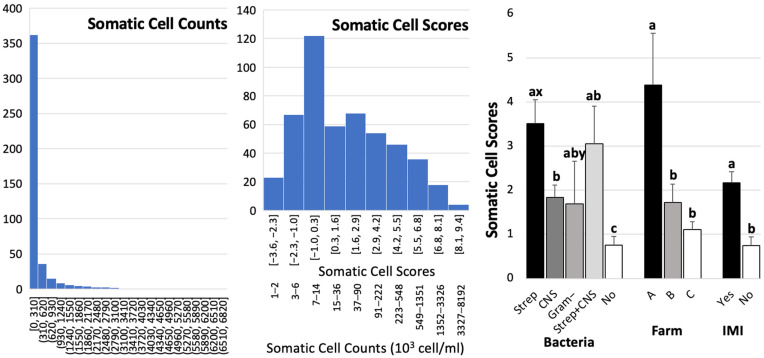
Data distribution of somatic cell count in 10^3^ cells/mL (left) and somatic cell scores (middle). The right figure compares SCS by type of bacteria, farm, and intramammary infection (IMI), where “Yes” indicates samples with bacteria and “No” indicates samples without bacteria. ^a, b, c^ indicate differences at *p* < 0.05; ^x, y^ ndicate differences at *p* < 0.1.

After excluding the trimmed data (approximately 13 to 15 data points), the final number of data points was 437 for protein, lactose, solid-not-fat, and total solids; 436 for milk fat; and 435 for freezing points, respectively. The largest dataset (*n* = 437) included data from 3 cows and 11 quarters from Farm A, 25 cows and 83 quarters from Farm B, and 213 cows and 343 quarters from Farm C. Overall means ± SEM for fat, protein, lactose, SNF, TS, and FP were 1.66 ± 0.03%, 3.25 ± 0.02%, 4.66 ± 0.16%, 8.71 ± 0.03%, 10.41 ± 0.04%, and −0.50 ± 0.002 °C, respectively. From the total number of samples (*n* = 450), only 377 SCC datasets were retrieved from the automated cell counter. The distributions of SCC and SCS are shown in Figure 2. SCCs ranged from 1 to 6719 cells/mL, with a median of 37 cells/mL. Pearson correlation coefficients among milk compositions and SCS are shown in Table 1. Most compositions and SCS were significantly correlated, except for fat–protein, protein–lactose, fat–SNF, and TS–SCS. Freezing points were negatively correlated with %protein, %lactose, SNF, and TS, and were positively correlated with %fat and SCS. Somatic cell scores were positively correlated with %milk fat and FP, but were negatively correlated with %protein, %lactose, and SNF.

The bacterial identification results show that the IMI for Farms A, B, and C were 90.91%, 45.45%, and 37.78%, respectively (Table 2). The most common bacteria found on all farms were CNS. A total of 11 mixed bacteria—all from CNS and Streptococci—were identified, with counts of 2, 8, and 1 for Farms A, B, and C, respectively. A significant association was found between the bacterial group and the farm.

**Figure 3:**
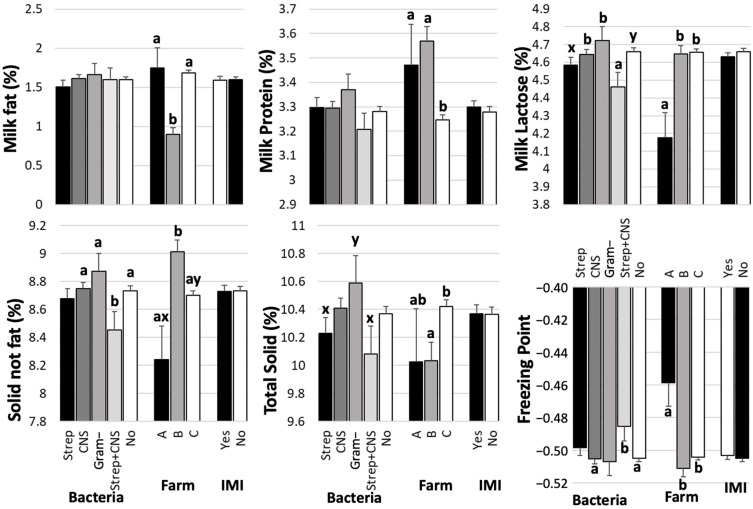
Means and standard error of the means (SEMs) of milk compositions among farms (Farm A, B, and C), bacteria (Streptococci (Strep), coagulase-negative staphylococci (CNS), Gram-negative bacteria (Gram -), streptococci and CNS (Mixed), none (No)), and infection status (Yes, with at least one bacterium found; No). ^a, b^ indicate significant differences at *p* < 0.05, and ^x, y^ indicate the tendency of differences at *p* < 0.1. The significance levels were determined using the least squares mean calculation of the simple mixed linear regression.

Comparisons of milk composition (Figure 3) and SCS (Left, Figure 2) among groups of bacteria, farms, and IMI are presented. The farm was associated with all milk compositions and SCS (*p* < 0.05). Although Farm A had a small sample size (*n* = 11), its milk quality relating to mastitis, as indicated by SCS, was significantly lower than that of Farms B (*p* = 0.03) and C (*p* = 0.006) (Figure 2, Left). Farm A had the highest average milk fat and freezing point, and the lowest lactose, SNF, and TS. Farm C demonstrated superior milk quality, as evidenced by a lower SCS compared to Farm A (*p* = 0.006). Additionally, the milk fat content was significantly higher at Farm C compared to Farm B at *p* < 0.0001. However, the levels of milk protein were significantly lower at Farm C than at Farm B (*p* < 0.0001). Furthermore, the lactose content at Farm C exceeded that at Farm A (*p* = 0.0009), while the total solids (TS) content was higher than that at Farm B (*p* = 0.0065).

No significant difference was observed between milk with and without bacterial identification, as indicated by the IMI parameter comparison among milk composition (Figure 3), except for SCS (Figure 2, left). The means (SEMs) of SCS and SCC were 2.17 (0.25) and 56 (14) × 10^3^ cells/mL for IMI milk and 0.74 (0.20) and 21 (14) × 10^3^ cells/mL for non-IMI milk, respectively. The ranges and medians of the SCC were 1 to 2567 and 45 × 10^3^ cells/mL for IMI milk and 1 to 4524 and 16 × 10^3^ cells/mL for non-IMI milk. Regarding bacterial groups, the SCS of milk with streptococci was higher than that of milk with CNS (*p* = 0.0056) and non-IMI milk (*p* < 0.0001). Milk with a mixed IMI of streptococci and CNS had significantly lower lactose and SNF levels than milk with CNS alone at *p* = 0.0278 and *p* = 0.0265, respectively, and with Gram-negative bacteria at *p* = 0.0235 and *p* = 0.0238, respectively (Figure 3). In contrast, the freezing point of the mixed IMI was higher than that of CNS (*p* = 0.026) and non-IMI milk (*p* = 0.0318).

## 4. Discussion

Regardless of the pathogens, most IMI milk composition parameters, except for SCS, were not significantly different from those of normal milk (Figure 3). A recent study also showed that bacterial contamination separated into sources of infection—for example, environmental and contagious pathogens—did not appear to significantly affect the yield of milk or its main components; namely, protein, casein, lactose, fat, TS, solid-not-fat, free fatty acid, or citric acid [22]. In contrast, our results revealed differences in certain milk compositions, including percentages of lactose, SNF, TS, and the milk’s freezing point, among bacterial groups, including streptococci, CNS, Gram-negative bacteria, and mixed IMI (Figure 3). This deviation in results may have been caused by differences in the definitions of bacterial groups, as Gram-negative bacteria—including streptococci—were separated in this study. Additionally, the limited number of streptococci in the study by Zalewska and colleagues [22] may limit the significance of this finding. Mastitis is known to lower the fat and lactose contents of milk [21,23,24] by reducing de novo synthesis of milk components caused by the inflammatory response, resulting in an incursion of blood components into milk. Our study did not show the effects of mastitis on milk fat content. This may have been due to the low fat content, with an average of 1.66 ± 0.03% in our study, as shown in Figure 1. The fat content typically ranges from 3.5% to 4.5% in cows without udder infections, with a slightly lower value of 2.8% in the tropical dairy industry [25], depending on the rearing system [26]. Limitations in facilities and farm management knowledge in a developing dairy industry may contribute to deviations in milk composition due to mastitis [11]. In addition, the milk samples in this study may refer to cisternal milk, which is milk collected after discarding foremilk prior to the release of alveolar milk [27]. Milk fat concentration increases markedly throughout milk letdown, with the lowest-fat milk drawn first and the highest-fat milk drawn last [28]. Lactose was negatively associated with SCS (Table 1), but lactose levels fluctuated among bacterial groups (Figure 3). The levels of immune response stimulation among pathogens also result in differences in the production of inflammatory cytokines, such as tumor necrosis factor-alpha (TNF-α), which affects lactose synthesis [29]. A primary mechanism for lactose reduction is the cytokine-mediated disruption of the blood–milk barrier; TNF-β, in particular, has been shown to compromise the integrity of epithelial tight junctions [30]. Furthermore, inflammatory cascade, including the massive influx of neutrophils recruited by cytokines, causes direct apoptotic and necrotic damage to milk-producing epithelial cells, which irreversibly reduces the gland’s total lactose-synthesizing capacity [31].

The present study demonstrated that IMI with CNS alone caused little deviation in milk composition compared with non-infected quarters [8], whereas mixed infections with Streptococci resulted in more pronounced changes (Figure 3). Specifically, the mixed IMI group showed significantly lower percentages of lactose and SNF, higher SCS, and elevated freezing points compared with CNS or Gram-negative infections. These findings suggest that although CNS are generally characterized as minor pathogens, their role in coinfections was supported by the exacerbated inflammatory responses [13,32] and substantially impaired milk quality, as shown in goat milk [14]. The decreases in lactose and SNF observed in the mixed IMI group are consistent with previous findings that mastitis reduces lactose synthesis and alters nutrient transport in the mammary epithelium [33]. The stronger effect of mixed infections suggests a possible synergistic interaction between Streptococci and CNS, resulting in more severe epithelial damage and impaired milk secretion [8]. Such interactions may also explain the higher freezing point observed, which could reflect alterations in milk osmolarity due to inflammation-related shifts in solute balance.

Our findings on SCS further support the notion that Streptococci are major drivers of somatic cell recruitment into infected quarters. After bacterial invasion, leukocytes and epithelial cells in infected quarters release chemoattractant products, facilitating the rapid movement of neutrophils into the infected areas [34], which causes an increase in the SCC. If the bacteria are destroyed, the recruitment of neutrophils into the gland ceases and only a mild inflammatory episode will be required to restore health in the gland [35]. In this study, while CNS infection alone was associated with lower SCS compared to Streptococcal infections, coinfections with Streptococci produced SCS values similar to or higher than those of Streptococci alone. This highlights the clinical importance of CNS when combined with other pathogens, as the inflammatory response may be intensified, contributing to reduced milk quality and potential economic losses. In general, SCC was more influential on major mastitis indicators, suggesting that automatic milking system sensors could be modified to monitor milk before ejection for more efficient mastitis detection [36].

Farm differences in milk composition and SCS observed in this study underline the impact of management practices on udder health and milk quality. Farm C, with lower SCS and fewer infected quarters, exhibited better milk quality traits, suggesting that better feeding and mastitis control strategies can mitigate the negative effects of IMI. Milk composition is influenced by both intrinsic and extrinsic factors related to the cow. In this study, lower fat content resulted in a lower TS content but not a lower SNF content, compared to the normal values reported for Iranian dairy cows at 11.79% and 9.02%, respectively [37]. Almost all milk contents were significantly correlated, except for fat–protein, protein–lactose, and fat–SNF. This suggests that almost all correlations found are related to the same source of nutrients for milk production, while some components, such as lactose and protein, are included in other parts, like SNF. Some sources of milk component biosynthesis derive from blood circulation; specifically, triglycerides and glucose for the synthesis of milk fat and lactose [38]. For extrinsic factors, milk composition and quality are influenced by cow factors, including animal breed, stage of lactation, parity [37], and management practices, such as feed and feeding systems [39,40]. Regarding the cow factors, it is well established that the composition of bovine milk is not static but rather fluctuates significantly throughout the lactation stage [41]. Concentrations of milk fat and protein exhibited a curvilinear pattern, characterized by high levels in early lactation (colostrum/transition milk), a decline to a minimum around 50–90 days in milk, and an increase during mid- and late lactation as milk yield declined [42]. The cows in mid- and late lactation, which were the most commonly used cows in this study, may have limited representativeness for the broader population in this group. Our study also shows that farm management was related to milk composition, as the means of fat, protein, lactose, SNF, and TS differed among farms. Differences in feeding management, especially for the highest feed quality, the highest milk production, and the lowest mastitis incidence, as shown in Table 2 for Farm C, may have resulted in the highest TS, with significantly higher fat than at Farm B and significantly higher lactose than at Farm A. In addition, the low SCS and low number of infected quarter milk samples at Farms B and C may also be related to the milk contents. The freezing point of milk is also influenced by factors related to variations in the environment, management, and breed [43]. Our results show that FP differed between farms, supporting the relationship between FP and farm management. The highest FP found in the mixed IMI was significantly higher than in the CNS alone and the no-infection groups, which may be related to changes in milk components after infection or the high severity of the mixed IMI. Our previous findings demonstrate that mixed infection increases the levels of cytokines [13] and exacerbates the clinical severity of mastitis in mice [32].

This study employed a cross-sectional design, which is suitable for identifying associations rather than establishing causation [44]. It is therefore essential to separate descriptive interpretation from mechanistic speculation. Determining the direction of this relationship would require a longitudinal study to track the temporal sequence of infection. The use of bacterial groups in this study may be a key constraint as pathogenicity, biofilm-forming ability, and antimicrobial resistance, among other factors, vary significantly among bacterial species within the group, such as CNS [7]. Additionally, our statistical model could not account for all potential unmeasured confounders, such as specific farm-management practices or environmental factors, which may influence the risk of coinfection. Future implications, such as stratified analysis, weighting, or multiple regression, are necessary for greater precision and leverage.

## 5. Conclusions

This study found that mixed infections of CNS and Streptococci cause more severe changes in milk composition than single-pathogen infections. This highlights the importance of CNS in coinfections, suggesting they should not be considered minor pathogens in udder health management. Further research with larger datasets and experimental studies is necessary to investigate and explore the mechanisms underlying these pathogen interactions and their comprehensive impacts on milk quality.

## Figures and Tables

**Table 1:** Pearson correlation coefficients among quarter milk compositions, including fat, protein, lactose, SNF, TS, somatic cell score (SCS, the linear score of SCC), and freezing point (FP). *, **, *** indicate the significant levels at *p* < 0.1, *p* < 0.05, and *p* < 0.01, respectively.

	Fat	Protein	Lactose	SNF	TS	FP
Protein	0.016					
Lactose	−0.107 **	0.024				
SNF	−0.080 *	0.704 ***	0.693 ***			
TS	0.732 ***	0.515 ***	0.353 ***	0.616 ***		
FP	0.107 **	−0.264 ***	−0.852 ***	−0.842 ***	−0.481 ***	
SCS	0.120 **	−0.178 ***	−0.520 ***	−0.291 ***	−0.083	0.500 ***

**Table 2:** Number (percentages of column) of bacteria groups in raw quarter milk among farms after exclusion of trimmed values of milk compositions (*n* = 437). A significant association was found between the bacterial group and the farm, as determined by Fisher’s exact chi-square test.

Bacteria Group	Farm			Total
	A	B	C	
Streptococci	0 (0)	12 (13.64)	23 (6.55)	35 (7.78)
Coagulase-negative Staphylococci (CNS)	8 (72.73)	19 (21.59)	88 (25.07)	115 (25.56)
Gram-bacteria	0 (0)	1 (1.14)	8 (2.28)	9 (2.00)
Streptococci-CNS (Mixed)	2 (18.18)	8 (9.09)	1 (0.28)	11 (2.44)
None detected (None)	1 (9.09)	47 (54.55)	225 (65.81)	280 (62.22)
Total	11	88	351	450

## Data Availability

The data presented in this study are available on request from the corresponding author.
